# *Euphorbia characias* Extract: Inhibition of Skin Aging-Related Enzymes and Nanoformulation

**DOI:** 10.3390/plants11141849

**Published:** 2022-07-14

**Authors:** Francesca Pintus, Sonia Floris, Antonella Fais, Benedetta Era, Clara Porcedda, Carlo Ignazio Giovanni Tuberoso, Carla Caddeo

**Affiliations:** 1 Department of Life and Environmental Sciences, University of Cagliari, SS 554-bivio per Sestu, 09042 Monserrato, Italy; fpintus@unica.it (F.P.); s.floris@unica.it (S.F.); fais@unica.it (A.F.); era@unica.it (B.E.); tuberoso@unica.it (C.I.G.T.); 2 Department of Biomedical Sciences, University of Cagliari, SS 554-bivio per Sestu, 09042 Monserrato, Italy; clara.porcedda@unica.it; 3 Department of Life and Environmental Sciences, University of Cagliari, Via Ospedale 72, 09124 Cagliari, Italy

**Keywords:** *Euphorbia* *characias*, phospholipid vesicles, nanoformulation, enzyme inhibition, skin aging, melanogenesis

## Abstract

Plant extracts have long served as important sources of bioactive compounds, and they are currently the focus of extensive research in the development of novel preventive and therapeutic strategies. However, their health benefits are often limited by low bioavailability. Nanoparticle delivery systems can represent a solution to such limitations. *Euphorbia characias* is a Mediterranean shrub known to have biological activities, such as inhibiting tyrosinase and showing a potential role as a skin-whitening agent. In this study, an ethanolic extract from *E. characias* leaves was tested for its inhibitory activity on skin-related enzymes, such as elastase, collagenase, and hyaluronidase, and for sun protection factors. Moreover, the extract was formulated in phospholipid vesicles to improve its local bioavailability and applicability. The vesicles were characterized by size, surface charge, storage stability, and entrapment efficiency. The nanoformulation was also evaluated for antioxidant activity and assayed for cytocompatibility and anti-tyrosinase activity in melanoma cells. Our findings demonstrated that the extract has a photo-protective effect and enzyme-inhibitory properties. *E. characias* nanoformulation was also cytocompatible and improved the extract’s activity in the cells, suggesting a potential skin application for antimelanogenic treatments and confirming the key role of nanotechnological approaches to maximize plant extract’s potentialities.

## 1. Introduction

*Euphorbia characias* belong to the Euphorbiaceae family containing latex inside specialized cells called laticifers. *E. characias* latex has been characterized in terms of its enzymatic properties, but its aerial parts are responsible for most of the biological activities reported up to now [1]. In particular, an ethanolic leaf extract possessed antimelanogenic properties, inhibiting tyrosinase activity in both cell-free and cellular systems [2]. Tyrosinase (EC 1.14.18.1) is the rate-limiting enzyme in the melanin synthesis process. It catalyzes the hydroxylation of l-tyrosine to l-DOPA (3,4-dihydroxyphenilalanine) and the subsequent oxidation of l-DOPA to *o*-dopaquinone. The subsequent steps involve oxidation and polymerization reactions to obtain melanin polymers. Even though melanin has an important role in protecting the skin from the toxic effects of UV rays, an excess in its production is correlated to hyperpigmentation-related disorders. Typical age spots are one of the major changes due to an increased tyrosinase activity and melanin production associated with aging. Thus, tyrosinase inhibitors have become increasingly important since they may be used as skin-whitening agents for treating skin-aging disorders.

Another characteristic of skin aging is the loss of structure in the extracellular matrix (ECM). The ECM comprises numerous proteins, including collagen and elastin, that play a major role in retaining skin elasticity. The degradation of ECM is mainly due to the enhanced activity of proteolytic enzymes, such as elastase, collagenase, and hyaluronidase. The inhibition of these enzymes by plant compounds might be a promising approach for preventing skin aging [3]. Elastase (EC 3.4.21.36) is a proteolytic enzyme involved in the physiological degradation of elastin, the protein responsible for skin elasticity. Increased elastase activity was found in several diseases, such as psoriasis, dermatitis, inflammatory processes, and premature skin aging with the associated formation of wrinkles. Collagenase (EC 3.4.24.3) belongs to the family of matrix metalloproteinases and can degrade the triple-helical region of collagen under physiological conditions. Collagen is the fibrous component of the ECM and the major structural protein in human skin; it provides structural support for bones, tendons, ligaments, and blood vessels. Hyaluronidase (EC 3.2.1.35) is a mucopolysaccharase that hydrolyzes glycosaminoglycans, including hyaluronic acid, in the EMC during tissue remodelling. When hyaluronidase activity is increased, such as during aging, the level of hyaluronic acid decreases, and the moisture and tension of the skin are reduced. Thus, inhibitors of the above enzymes could be useful as anti-wrinkle and anti-aging agents.

Plant-derived products are currently the focus of extensive research due to their numerous biological activities [4,5]. However, the health benefits are often limited by the low bioavailability of bioactive compounds due to their high instability, low aqueous solubility, and/or poor absorption. Nanoparticle delivery systems can represent a solution to such limitations, as they can entrap and transport a variety of bioactive compounds, providing protection, controlled release, enhanced efficacy, and reduced toxicity [6,7]. With regards to skin applications, phospholipid vesicles have been extensively studied. To overcome the skin barrier layer and promote active molecules penetration, several approaches have been proposed. Over the last three decades, increasing attention has been given to altering the properties of these vesicles by adding chemical penetration enhancers (e.g., surfactants, ethanol) that interact with skin constituents to enhance drug flux [8]. The literature describes transfersomes, ethosomes, and PEVs (Penetration Enhancer-containing Vesicles), which differ in terms of structure and composition, but share the ability to penetrate deeper into the skin with their cargo [9].

In this study, an ethanolic extract of *E. characias* was tested for inhibitory activity against aging-related enzymes. The extract was then formulated in PEVs to improve its bioavailability and applicability to the skin. The vesicles were characterized by size, surface charge, storage stability, entrapment efficiency, and antioxidant activity. Furthermore, the cytocompatibility and anti-tyrosinase activity were assayed in melanoma cells.

## 2. Results

### 2.1. Quali-Quantitative Determination of Phenolic Compounds in E. characias Extract by HPLC-DAD Analysis

**Figure 1:**
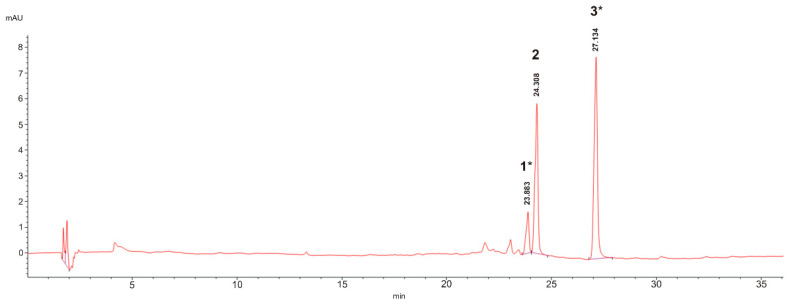
HPLC-DAD chromatogram of *E. characias* extract at λ = 360 nm. Chromatographic conditions are described in the text. 1: quercetin-3-O-arabinoside; 2: quercetin-3-O-rhamnoside; 3: quercetin-3-(2-O-acetyl)-arabinoside; * tentative attribution and amount expressed as quercetin-3-O-rhamnoside equivalents.

The HPLC–DAD qualitative evaluation of the major phenolic compounds in the extract from *E. characias* leaves confirmed the typical composition of the extract, as previously reported by Pisano et al. [10]. The most abundant polyphenolic compounds were flavonoids, and, for this reason, the analysis was performed at λ = 360 nm (Figure 1). Quercetin-3-O-rhamnoside (peak 2) was previously identified in *E. characias* leaf extract [10], and it was confirmed by comparison with a pure standard. Peaks 1 and 3 were tentatively identified as quercetin-3-O-arabinoside and quercetin-3-(2-O-acetyl)-arabinoside by comparison with HPLC-DAD data and UV-VIS spectra from both our previous characterization of the same *E. characias* population [10] and the literature data reported for *E. characias* extracts [11,12,13]. The calibration curve built for quercetin-3-O-rhamnoside was used for the quantitative analysis of the three flavonols. Quercetin-3-(2-O-acetyl)-arabinoside was the most abundant compound (28.59 ± 0.03 mg/g dry extract) followed by quercetin-3-O-rhamnoside and quercetin-3-O-arabinoside (18.83 ± 0.06 and 5.15 ± 0.02 mg/g dry extract, respectively).

### 2.2. Biological Activities

The ethanolic extract from *E. characias* leaves was previously found to exert several biological activities [1]. Among them, we have described tyrosinase inhibition in both cell-free and cellular systems [2]. Tyrosinase activity is enhanced during aging, as well as that of other enzymes, causing a degradation of the ECM and a consequent structural fragility, loss of elasticity, and wrinkle formation.

In this study, the *E. characias* extract was tested to evaluate the potential inhibition of aging-related enzymes elastase, collagenase, and hyaluronidase. The IC_50_ values obtained from the extract were compared with those of standard inhibitors in order to evaluate the inhibitory strength of the extract (Table 1). The extract exerted inhibitory activity toward all the tested enzymes. The strongest activity was observed against hyaluronidase, with an IC_50_ value about three-fold lower than that of the standard inhibitor, oleanolic acid.

For extracts with potential skin application, it could be important to determine the sun protection factor (SPF). The SPF indicates the ability to absorb UV rays, protecting the skin from the toxic effects produced by such radiation. Indeed, UV rays are responsible for skin disorders, and they trigger the processes that result in skin aging, oxidative stress, and wrinkle formation. *E. characias* extract displayed a good photo-protective effect, with an SPF value of 9.10 at the concentration of 100 µg/mL (Table 2). The reduction in the absorption of UV radiation enhances, in an indirect way, the antioxidant activities and the inhibition of aging-related enzymes.

### 2.3. Vesicle Characterization

This study’s aim was to produce a nanoformulation that would safely and effectively deliver the *E. characias* extract’s bioactive compounds. Particularly, a potential topical application of this formulation for the treatment of age-related skin disorders was supposed.

Et-PEVs, which are phospholipid vesicles modified with ethanol (Et) to promote skin penetration, were produced and characterized in terms of size, homogeneity, and surface charge. To evaluate the extract’s effect on the vesicles, the *E. characias* Et-PEVs were compared with the empty Et-PEVs. The light scattering results, as reported in Table 3, showed that the empty vesicles were 85 nm in diameter, homogeneously dispersed (PI 0.28), and highly negatively charged (−68 mV). The extract’s incorporation significantly increased the mean diameter of the vesicles, although they remained small (around 100 nm), whereas the polydispersity index and zeta potential values were unaltered (Table 3).

The stability of the nanoformulations was evaluated by monitoring the mean diameter, the polydispersity index, and the zeta potential during a 90 day-storage period at 4 °C. No significant alterations were detected.

The entrapment efficiency of the Et-PEVs was calculated based on the amount of three quercetin derivatives identified in the *E. characias* extract and detected in the dialyzed and non-dialyzed vesicle dispersions. The Et-PEVs entrapped high amounts of the extract; the entrapment efficiency was over 85% for the three compounds considered (Table 3).

### 2.4. Antioxidant Activity

The antioxidant activity (AA) of the *E. characias* extract was estimated as a function of the ability to scavenge DPPH radicals (Table 4). The solution of *E. characias* extract (2 mg/mL) scavenged the DPPH completely (AA ~96%; 374 μg/mL of Trolox equivalents). Such strong activity was not affected by the incorporation of the extract in the Et-PEVs, as demonstrated by the unaltered values of AA and TE (*p* > 0.05; Table 4).

Given the presence of phospholipids, the empty Et-PEVs exerted a slight antioxidant activity (AA ~18%; Table 4).

### 2.5. Cell Viability and Cellular Tyrosinase Inhibition

**Figure 2:**
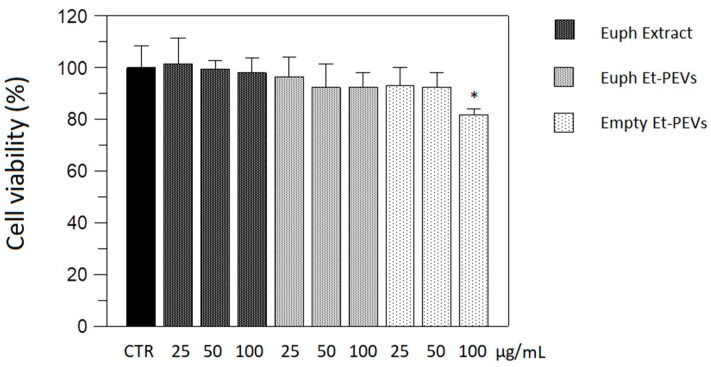
Effect of *E. characias* extract, in ethanol solution and in Et-PEVs, and empty Et-PEVs, on the viability of B16F10 melanoma cells. * indicates statistical difference vs CTR (*p*-value = 0.03).

We examined whether the *E. characias* nanoformulations exerted an antimelanogenic effect by inhibiting tyrosinase in cells. First, we assessed the safety of the extract, free and nanoformulated, by evaluating the effects on the viability of B16F10 melanoma cells. The effect of empty Et-PEVs was also determined. After exposing the cells for 48 h to various concentrations of the samples, the viability was examined using an MTT test. The results showed that cell viability was not negatively affected, and it was still around 82% (*p* < 0.05 vs. control cells, CTR) when the higher concentration of empty Et-PEVs was tested. This indicates that none of the samples can be considered cytotoxic (Figure 2).

**Figure 3:**
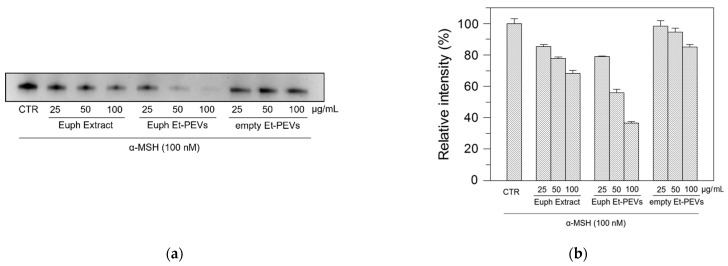
Tyrosinase zymography. (**a**) Effect of *E. characias* extract, in ethanol solution or in Et-PEVs, and empty Et-PEVs on cellular tyrosinase activity estimated by L-DOPA staining; (**b**) relative intensity of bands determined with ImageJ software. Empty Et-PEVs at the concentration of 25 μg/mL are not statistically different from the CTR, while different at 50 μg/mL (*p*-value = 0.02). All the other samples are statistically different from each other at equal concentration and from CTR with *p*-value < 0.01.

To investigate the anti-melanogenic effect, the cellular tyrosinase activity was estimated by zymography. An l-DOPA staining assay was carried out with lysates of α-melanocyte-stimulating hormone (α-MSH)-stimulated B16F10 cells treated with or without *E. characias* samples. Figure 3 clearly shows that upon incubation with the extract, free or nanoformulated, the activity of tyrosinase decreased, and lighter bands were observed. The effect of the empty Et-PEVs was negligible as compared to that of the samples containing the extract. Interestingly, the *E. characias* Et-PEVs exerted a higher dose-dependent tyrosinase inhibition compared to the extract ethanol solution (*p* < 0.01 for all the concentrations), suggesting that the incorporation of the extract results in a greater antimelanogenic effect in a cellular system.

## 3. Discussion

*E. characias* leaf extract possesses several biological activities, such as the inhibitory effect on tyrosinase, previously described in both cell-free and cellular systems. Tyrosinase is implicated in skin aging processes, along with elastase, collagenase, and hyaluronidase, and proteolytic enzymes, whose increased activity can lead to the degradation of ECM. Other plant extracts were demonstrated to inhibit these enzymes. A leaf extract from *Stenocarpus sinuatus* was found to have anti-aging activity owing to anti-collagenase, anti-elastase, anti-tyrosinase, and anti-hyaluronidase activities [14]. The same inhibitory properties were shown by extracts from four southern African medicinal plants, *Clerodendrum glabrum, Schotia brachypetala, Psychotria capensis*, and *Peltophorum africanum* [15]. Various plant extracts have also shown antimelanogenic activity [16]. An extract from *Euphorbia supina*, a plant belonging to the Euphorbiaceae family, reduced tyrosinase activity and melanin content in a dose-dependent manner in B16F10 cells [17]. Salem et al. [18] investigated the anti-aging activity of extracts from five medicinal plants belonging to phenolic-rich families, namely *Rosmarinus officinalis*, *Lavandula officinalis*, *Matricaria chamomilla*, *Camellia sinensis*, and *Pelargonium graveolens*, frequently used in the preparation of ethnomedicinal recipes for the prevention or treatment of aging. The results showed that *R. officinalis* had the highest total phenolic content, which was correlated with its potent antioxidant and anti-aging activities.

In this study, we confirmed that the inhibition of the above enzymes by *E. characias* may be a promising approach in the treatment of age-related skin disorders. Comparing the effect of the *E. characias* extract to that of standard inhibitors, it exerted less of an effect on elastase, showing an IC_50_ about three-fold higher than the standard inhibitor, oleanolic acid. An effect comparable to that of epigallocatechin gallate was instead observed for collagenase. The best inhibitory activity was detected against hyaluronidase since the extract shows a three-fold higher inhibition compared with the standard compound. Considering the overall activities tested, even if with different efficacy, the extract is active on all of the four aging-related enzymes. Moreover, *E. characias* extract showed a good photo-protective effect by reducing the absorption of UV radiation. UV rays are responsible for many skin diseases, and they trigger the processes that result in skin aging, oxidative stress, and wrinkle formation by ROS production and the activation of tyrosinase and ECM-degrading enzymes. The reduction in the absorption of this radiation, therefore, enhances the effect of the extract by increasing, in an indirect way, the antioxidant activities and the inhibition of aging-related enzymes.

Furthermore, a nanoformulation was produced to deliver the *E. characias* extract safely and effectively to the skin. *E. characias* Et-PEVs displayed a small size, high entrapment efficiency, and long-term stability, which are ideal requirements for cutaneous application. In addition, Et-PEVs preserved the antioxidant properties of the extract and exerted a higher tyrosinase inhibition compared to the free extract. All these findings suggest that the antimelanogenic effect of *E. characias* extract was potentiated by the incorporation in the vesicles. These achievements could be a starting point to investigate the potential improvement of other activities of *E. characias* extract, such as the wound healing activity. A previous study [12] showed that *E. characias* was useful for the healing of wounds and highlighted that compounds such as quercetin-3-O-rhamnoside and quercetin-3-O-arabinoside contributed to this activity. These compounds are contained in our extract as well; therefore, the evaluation of the wound healing potential of *E. characias* Et-PEVs is worthy of investigation. To the best of our knowledge, this is the first study that reports the nanoformulation of an extract from *E. characias* and the assessment of its biological activities in cells. Further investigation is needed to achieve a full picture of the multiple bioactivities of the extract and their enhancement upon nanoformulation, both in cells and in animal models of age-related skin disorders.

## 4. Materials and Methods

### 4.1. Materials

Lipoid S75 (S75), a mixture of soybean phospholipids (70% phosphatidylcholine, 9% phosphatidylethanolamine, and 3% lysophosphatidylcholine), triglycerides, and fatty acids, was purchased from Lipoid GmbH (Ludwigshafen, Germany). Ethanol, UPLC-gradient grade acetonitrile, methanol, 85% *w*/*w* phosphoric acid, 2,2-diphenyl-1-picrylhydrazyl (DPPH), and all the other reagents, if not otherwise specified, were purchased from Sigma-Aldrich/Merck (Milan, Italy). Standard quercetin-3-*O*-rhamnoside was purchased from Extrasynthese (Genay Cedex, France). Ultrapure water (18 MΩ·cm) was obtained with a Milli-Q Advantage A10 System (Millipore, Milan, Italy).

### 4.2. Extract Preparation

A voucher specimen for *E. characias* was deposited in the Department of Life and Environmental Sciences, University of Cagliari, Italy (number 1216/16 Herbarium CAG). Leaves of *E. characias* were collected at several locations in southern Sardinia (Italy), immediately frozen at −80 °C and lyophilized. The extraction was carried out in the dark at room temperature for 24 h under continuous stirring, according to a procedure previously reported [2]. Before use, 1 mg of dried powder was dissolved in 10% ethanol (1 mL).

### 4.3. Vesicle Preparation and Characterization

The *E. characias* extract was weighed in a glass vial and dissolved in ethanol; thereafter, S75 and water were added (Table 5). To obtain Et-PEVs, the dispersion was sonicated (3 s on and 2 s off, 10 cycles; 13 microns of probe amplitude) with an ultrasonic disintegrator (Soniprep 150 plus; MSE Crowley, London, UK).

For comparative purposes, empty Et-PEVs (i.e., without *E. characias* extract) were also prepared (Table 5).

The mean diameter, polydispersity index (PI), and zeta potential of the Et-PEVs were determined by dynamic and electrophoretic light scattering using a Zetasizer nano-ZS (Malvern Panalytical, Worcestershire, UK). The samples (*n* > 10) were diluted with water (1:100) and analyzed at 25 °C.

The above three parameters were monitored for 90 days to assess the long-term stability of the nanoformulations.

The non-incorporated extract components were removed from the Et-PEVs dispersions through dialysis. A 1 mL sample was loaded into Spectra/Por^®^ tubing (12,000–14,000 Da MWCO; Spectrum, DG Breda, The Netherlands) and kept in water (2 L), under gentle stirring, for 2 h. Non-dialyzed and dialyzed vesicles were disrupted by diluting (1:100 *v*/*v*) with methanol and analyzed by HPLC–DAD to determine the amounts of targeted phenolic compounds (see Section 4.4). The entrapment efficiency (E) was calculated as the percentage of the flavonols detected in dialyzed vs non-dialyzed Et-PEVs.

### 4.4. HPLC–DAD Analysis

The identification and quantification of phenolic compounds in the *E. characias* extract were performed by HPLC-DAD analysis, as described by De Luca et al. [19], using a 1260 Infinity II HPLC system (Agilent Technologies, Cernusco sul Naviglio, Milan, Italy). A Kinetex EVO C18 column (150 × 4.6 mm, 2.6 μm, Phenomenex, Casalecchio di Reno, Bologna, Italy) was used, with a mobile phase consisting of 0.22 M phosphoric acid and acetonitrile mixed with gradient elution. The chromatograms and spectra were elaborated with an OpenLab V. 2.51 data system (Agilent Technologies), and flavonols were detected and quantified at 360 nm [10]. The calibration curve for quercetin-3-*O*-rhamnoside was calculated by regression analysis by plotting the peak area obtained after standard injection (3 replicates at each concentration) against known standard concentrations (correlation values = 0.9999). For the HPLC–DAD analysis, *E. characias* extract was diluted to 1:100 *w*/*v* with an 80:20 (*v*/*v*) methanol: 0.22 M phosphoric acid mixture, and Et-PEVs were injected after dilution with methanol (1:100 *v*/*v*; see Section 4.3). The solutions were filtered with a 0.45 μm cellulose acetate syringe filter before injection.

### 4.5. Biological Activities

For the enzymatic assays described below, the IC_50_ values, the concentration giving 50% inhibition of enzyme activities, were determined by the interpolation of dose–response curves. The data from the activity assays were recorded with an Ultrospec 2100 spectrophotometer (Biochrom Ltd., Cambridge, UK).

#### 4.5.1. Elastase Inhibition Assay

Elastase inhibition was determined by using the substrate N-succ-(Ala)3-nitroanilide (SANA), as previously described [20]. A reaction mixture containing porcine pancreatic elastase (3.3 μg/mL) in 0.1 M Tris-HCl buffer (pH 8.0) was incubated with or without the extract for 20 min. The enzyme activity, after the addition of the substrate (1.6 mM), was monitored following the release of *p*-nitroaniline at 410 nm. Oleanolic acid was used as a positive control.

#### 4.5.2. Collagenase Inhibition Assay

Collagenase from *Clostridium histolyticum* (1 U/mL) was prepared in Tricine buffer (50 mM tricine, 10 mM calcium chloride, 400 mM sodium chloride, pH 7.5); the solution was incubated with the extract at different concentrations for 15 min. After the addition of the synthetic substrate N-(3-[2-Furyl]-acryloyl)-Leu-Gly-Pro-Ala (FALGPA), prepared in the same buffer solution, up to the final concentration of 0.8 mM, the absorbance was monitored at 340 nm. Epigallocatechin gallate was used as a positive control.

#### 4.5.3. Hyaluronidase Inhibition Assay

A hyaluronidase inhibitory assay was performed following the method described by Chompoo et al. [3]. A test sample of 5 μL was pre-incubated in 100 μL of bovine hyaluronidase (1.5 U) containing 20 mM sodium phosphate buffer (pH 7.0), 77 mM sodium chloride, and 0.01% bovine serum albumin (BSA). After 10 min at 37 °C, 100 μL of the substrate solution (0.03% hyaluronic acid in 300 mM sodium phosphate, pH 5.35) was added to the reaction mixture, followed by incubation for 45 min at 37 °C. Undigested hyaluronic acid was precipitated with acid albumin solution (1 mL), containing 0.1% BSA in 24 mM sodium acetate and 79 mM acetic acid (pH 3.75). The solution was maintained at room temperature for 10 min, and the absorbance was measured at 600 nm. Oleanolic acid was used as a positive control.

#### 4.5.4. Determination of Sun Protection Factor

The sun protection factor (SPF) of *E. characias* extract was determined by using the UV absorbance method, as previously reported [20]. The absorbance of the extract (0.1 mg/mL) was measured in a 290–320 nm range, with 5 nm increments, and three determinations were made at each point. The SPF was calculated by applying the Mansur Equation [21]:

SPF = CF × Σ^320^_290_ × EE(λ) × I(λ) × Abs(λ)

where CF = correction factor (10); EE (λ), I (λ), and Abs (λ) are the erythemal action spectrum, solar intensity spectrum, and UV absorbance of the sample, respectively. The values of EE (λ) × I(λ) are constant, as reported in the literature and shown in Table 6. They were determined according to the method reported by Sayre et al. [22].

### 4.6. Antioxidant Activity: DPPH Assay

The antioxidant activity of *E. characias* extract in ethanol solution or in Et-PEVs (2 mg/mL), and empty Et-PEVs was assessed by evaluating the ability to scavenge DPPH radicals. A 25 μM DPPH methanol solution (2 mL) was mixed with 20 μL of each sample and incubated for 30 min at room temperature in the dark. Thereafter, the absorbance (Abs) was measured at 517 nm against blank. The decrease in absorbance was quantified as a direct indication of the ability to scavenge the DPPH radical. The latter was expressed both as percent antioxidant activity (AA) calculated according to Equation (1):

AA = (Abs_DPPH_ − Abs_sample_/Abs_DPPH_) × 100 (1)

and as Trolox Equivalents (TE). The TE values were calculated using a calibration curve plotted with a series of Trolox (reference standard) concentrations (12.5–500 μg/mL). The results were expressed as μg TE/mL of the solution. The TE values reflect the ability of antioxidants to scavenge DPPH radical as compared with Trolox: the higher the TE values, the higher the radical scavenging activity of the investigated sample.

### 4.7. Cell Culture

Murine melanoma B16F10 cells (CRL-6475) were obtained from the American Type Culture Collection (ATCC, Manassas, VA, USA). The cells were cultured in Dulbecco’s Modified Eagle Medium supplemented with 10% fetal bovine serum (Gibco, NY, USA) and 1% penicillin/streptomycin (Euroclone, Milan, Italy). The cells were incubated in a humidified atmosphere of 5% CO_2_ at 37 °C. Cell viability was examined by the colorimetric 3-(4,5-dimethylthiazol-2-yl)-2,5-diphenyltetrazolium bromide (MTT) assay. Briefly, the cells were seeded in a 96-well plate (10^4^ cells/well) and incubated with various concentrations of *E. characias* extract (25, 50, and 100 μg/mL). After 48 h, the cells were labelled with the MTT solution for 3 h at 37 °C. The resulting violet formazan precipitates were dissolved in isopropanol, and the absorbance of each well was determined at 590 nm using a microplate reader with a 630 nm reference.

### 4.8. Tyrosinase Zymography

To clarify the tyrosinase inhibitory effect of the *E. characias* extract on melanogenesis, we determined the intracellular tyrosinase activity in B16F10 cells according to the l-DOPA-staining assay [23]. The cells were treated with α-MSH alone, or α-MSH plus *E. characias* extract at different concentrations (25, 50, and 100 μg/mL). After 48 h, the cells were harvested with lysis buffer (50 mM phosphate buffer, pH 6.8, containing 1% Triton X-100 and 0.1 mM phenylmethyl-sulfonyl fluoride). Cellular lysates were centrifuged at 12,000 rpm for 20 min at 4 °C, and the Bradford method was used to determine the protein content of the supernatant [24]. The protein extracts (5 μg) were then resolved by 8% SDS-polyacrylamide gel electrophoresis without mercaptoethanol or heating. After running, the gel was washed in 0.1 M phosphate buffer (pH 6.8) for 30 min twice. The gel was stained after incubation for 1 h at 37 °C in the dark with l-DOPA solution (5 mM) in 0.1 M phosphate buffer (pH 6.8). Tyrosinase activity was visualized in the gel as dark melanin-containing bands.

### 4.9. Statistical Analysis

The results are reported as means ± standard deviations (SD). Statistical differences were evaluated using GraphPad Prism software version 8 (San Diego, CA, USA). The multiple comparison of groups was evaluated by means of a two-way ANOVA followed by Tukey’s multiple test to demonstrate the statistical differences between the groups; Dunnet’s test was used for comparison between groups and control. Values with *p* < 0.05 were considered significant.

## 5. Conclusions

The results of the present work highlighted that *E. characias* has promising anti-aging activities. An ethanolic leaf extract, in addition to exerting inhibitory activity on tyrosinase, inhibits other enzymes related to skin aging, such as elastase, collagenase, and hyaluronidase, and exerts a photo-protective effect. These effects are supported by a strong antioxidant activity that can be explained by the presence of polyphenols and especially flavonoids, which represent a large part of the extracted content. Moreover, the extract was successfully incorporated into phospholipid vesicles. The nanoformulation was able to deliver the extract into cells and increase the antimelanogenic effect without interfering with cell viability. Therefore, the potential use of a nanoformulation of *E. characias* extract can represent an important perspective in medical applications for skin disorders.

## Figures and Tables

**Table 1:** Collagenase, elastase, and hyaluronidase inhibition, expressed as IC_50_ values (μg/mL), of *E. characias* (Euph) extract. Standard compounds were epigallocatechin gallate for collagenase and oleanolic acid for both elastase and hyaluronidase.

	IC_50_ (μg/mL)		
	Collagenase	Elastase	Hyaluronidase
Euph extract	120.5 ± 8.5	45.4 ± 2.5	33.6 ± 2.1
Epigallocatechin gallate	120.8 ± 6.2	-	-
Oleanolic acid	-	11.8 ± 0.6	97.0 ± 4.2

**Table 2:** Absorbance and sun protection factor (SPF) values of *E. characias* ethanolic extract.

Wavelength (nm)	Absorbance
290	1.347
295	1.190
300	1.047
305	0.895
310	0.741
315	0.609
320	0.504
SPF	9.10

**Table 3:** Characteristics of empty and *E. characias* (Euph) Et-PEVs: mean diameter (MD), polydispersity index (PI), zeta potential (ZP), and entrapment efficiency (E). Each value represents the mean ± SD (*n* > 10).

Formulation	MD nm ± SD	PI	ZP mV ± SD	E % ± SD
empty Et-PEVs	85.0 ± 7.1	0.28 ± 0.05	−67.8 ± 11.1	--
Euph Et-PEVs	101.1 ± 9.7 *	0.29 ± 0.07	−70.6 ± 7.3	quercetin-3-*O*-arabinoside 86.2 ± 3.4 ^#^ quercetin-3-*O*-rhamnoside 85.6 ± 3.6 quercetin-3-(2-*O*-acetyl)-arabinoside 86.3 ± 3.1 ^#^

* value statistically different (*p* < 0.05) from empty Et-PEVs. ^#^ expressed as quercetin-3-O-rhamnoside equivalents.

**Table 4:** Antioxidant activity of the vesicle formulations in comparison with an ethanol solution of the *E. characias* extract (Euph). DPPH results are expressed as Antioxidant Activity (AA) and as Trolox Equivalents (TE). Results are reported as the mean value ± SD of 3 separate experiments, each performed in triplicate.

Formulation	AA % ± SD	TE μg/mL
empty Et-PEVs	18.4 ± 0.4	20 ± 6.1
Euph Et-PEVs	94.8 ± 0.4	370 ± 1.3
Euph solution	95.8 ± 0.6	374 ± 3.0

**Table 5:** Composition of the vesicle formulations.

Formulation	S75 ^1^	Euph ^2^	Et ^3^	H_2_O
empty Et-PEVs	120 mg	--	0.1 mL	0.9 mL
Euph Et-PEVs	120 mg	2 mg	0.1 mL	0.9 mL

^1^ soybean phospholipids; ^2^ *E. characias* extract; ^3^ ethanol.

**Table 6:** E (λ) and I (λ) values used for SPF calculation.

Wavelength (nm)	EE × I
290	0.0150
295	0.0817
300	0.2874
305	0.3278
310	0.1864
315	0.0837
320	0.0180

## Data Availability

Not applicable.
